# Underwater versus conventional endoscopic mucosal resection for ≥10 mm sessile or flat colorectal polyps: A systematic review and meta-analysis

**DOI:** 10.1371/journal.pone.0299931

**Published:** 2024-03-07

**Authors:** Xue Wang, Yue Wang, Xueyan Cao, Chunmei Zhang, Lin Miao

**Affiliations:** 1 Medical Centre for Digestive Diseases, The Second Affiliated Hospital of Nanjing Medical University, Nanjing, Jiangsu, China; 2 Emergency Department, Luzhou People’s Hospital, Luzhou, Sichuan, China; Hospital Universitario de Canarias, SPAIN

## Abstract

**Background and aim:**

Underwater endoscopic mucosal resection (UEMR) has been an emerging substitute for conventional EMR (CEMR). This systematic review and meta-analysis aimed at comparing the efficiency and safety of the two techniques for removing ≥10 mm sessile or flat colorectal polyps.

**Methods:**

PubMed, Cochrane Library and Embase databases were searched up to February 2023 to identify eligible studies that compared the outcomes of UEMR and CEMR. This meta-analysis was conducted on the en bloc resection rate, R0 resection rate, complete resection rate, procedure time, adverse events rate and recurrence rate.

**Results:**

Nine studies involving 1,727 colorectal polyps were included: 881 were removed by UEMR, and 846 were removed by CEMR. UEMR was associated with a significant increase in en bloc resection rate [Odds ratio(OR) 1.69, 95% confidence interval(CI) 1.36–2.10, *p*<0.00001, *I*^*2*^ = 33%], R0 resection rate(OR 1.52, 95%CI 1.14–2.03, *p* = 0.004, *I*^*2*^ = 31%) and complete resection rate(OR 1.67, 95%CI 1.06–2.62, *p* = 0.03, *I*^*2*^ = 0%) as well as a significant reduction in procedure time(MD ‒4.27, 95%CI ‒7.41 to ‒1.13, *p* = 0.008, *I*^*2*^ = 90%) and recurrence rate(OR 0.52, 95%CI 0.33–0.83, *p* = 0.006, *I*^*2*^ = 6%). Both techniques were comparable in adverse events rate.

**Conclusion:**

UEMR can be a safe and efficient substitute for CEMR in removing ≥10 mm sessile or flat colorectal polyps. More studies verifying the advantages of UEMR over CEMR are needed to promote its application.

## Introduction

Colorectal cancer (CRC) is the third most common cause of the cancer death worldwide. The confirmed cases and death cases of CRC in 2019 have added to 2.17 million and 1.09 million respectively [1]. The morbidity and mortality of CRC can be reduced through appropriate surveillance and screening. As a diagnostic and therapeutic tool, colonoscopy is the predominant screening means for CRC [2]. According to a 10-year follow-up population-based randomized trial, colonoscopy screening reduced the risk of CRC from 1.22% to 0.84%, and the risk of CRC-related death from 0.30% to 0.15% [3]. Therefore, colonoscopy screening and endoscopic resection technique are indispensable for CRC prevention.

Endoscopic mucosal resection (EMR) is the preferred technique to remove 10–20 mm sessile or flat colorectal polyps [4]. In the conventional EMR (CEMR) procedure, gas is used to fill and distend the lumen, and polyps are lifted by submucosal injection to create a cushion to avert complications, such as perforation. However, the resection of sessile or flat colorectal polyps is challenging, and CEMR cannot achieve complete resection in 100% of cases [5]. Additionally, en bloc resection in CEMR becomes difficult as the size of the polyp increases [6]. Thus, novel methods to improve en bloc resection rates and reduce residual lesion rates are clearly needed.

In 2012, underwater EMR (UEMR) was first proposed as an alternative technique to CEMR by Binmoeller et al. [7]. UEMR uses water instead of gas to dilate the lumen and resects polyps without submucosal injection. Multiple studies have demonstrated the favourable outcomes of UEMR in removing sessile or flat colorectal polyps. Yamashina et al. [8] concluded that UEMR greatly improved R0 resection rate for 10–20 mm polyps. Nagl et al. [6] reported that UEMR was superior to CEMR in en bloc resection rate and R0 resection rate for 20–40 mm polyps.

Up to now, the effectiveness of UEMR versus CEMR for resecting ≥ 10 mm sessile or flat colorectal polyps is still unclear. In this systematic review and meta-analysis, we included randomized controlled trials (RCTs) and cohort studies to objectively evaluate the efficiency and safety of UEMR and CEMR in these fields.

## Methods

### Literature search

PubMed, Cochrane Library and Embase databases were comprehensively searched up to February 2023 by two investigators to identify all relevant studies. The following words made up the search strategy: (((resection OR mucosectomy) AND endoscop*) OR “endoscopic mucosal resection” OR EMR) AND (underwater OR water) AND (colon* OR colorectal OR rectum OR rectal OR intestin*) AND (polyp* OR lesion* OR adenoma* OR neoplasm* OR tumor* OR tumour* OR carcinom* OR cancer*). The complete electronic strategies for the three databases are outlined in S1 Table.

We also reviewed the bibliographies of the included articles as well as meta-analysis conducted on the relevant topic for eligible studies that may not have been detected before. With regard to the same studies in different versions, the latest or more complete study was reserved. Disagreements were resolved via discussion.

The inclusion criteria were RCTs or cohort studies that compared UEMR and CEMR for removing ≥10 mm sessile or flat colorectal polyps. The exclusion criteria were studies with minor participants (<18 years of age), salvage for recurrent colorectal lesions, procedure protocol different from the normal EMR, nonhuman research and articles published not in English.

### Date extraction

Data of interest were extracted by two investigators independently into a standardized data extraction sheet, including first author name, year, country, study design, research duration, inclusive and exclusive criteria, number of patients and polyps in UEMR and CEMR, patient demographics, polyp size and location, and primary outcomes (procedure time, proportion of en bloc resection, R0 resection, complete resection, adverse events such as bleeding and perforation, follow-up duration and recurrence). For studies with different polyp morphologies and multiple lesion size groups, only data of ≥10 mm nonpedunculated colorectal polyps were eligible and extracted. The missing data were obtained by contacting the original authors via e-mail. Disagreements were settled by discussion until reaching a consensus.

### Definition and outcomes

The outcomes of interest were the en bloc resection rate, R0 resection rate, complete resection rate, procedure time, adverse events rate and recurrence rate. En bloc resection was defined as endoscopically integral removal of a polyp in one piece, with no remnant tissue visible on conventional white light imaging and narrow band imaging. R0 resection was defined as en bloc resection with tumor-free lateral and vertical margins. Complete resection refers to complete removal of the lesion without any adenomatous or serrated pathology. Procedure time referred to the period from the start of water immersion or lesion perimeter marking (UEMR) or submucosal injection (CEMR) to the complete removal of lesions or management of immediate complications or closure of mucosal defects with hemoclips. Intraprocedural bleeding, delayed bleeding and perforation constituted the adverse events in this meta-analysis. Intraprocedural bleeding refers to the bleeding lasting >30 seconds after resection and requires endoscopic intervention. Delayed bleeding refers to any bleeding occurring within 30 days after resection and requiring medical intervention. Perforation referred to visual peritoneal fat or extramural organ on the endoscopic image or pneumoperitoneum on computed tomography. Recurrence was defined as a new lesion that appears at the previous excision site during the period of endoscopic follow-up.

### Statistical analysis

The Mantel‒Haenszel test and inverse variance test were utilized to analyse dichotomous variables and continuous variables respectively. The analysis outcomes of dichotomous data are presented as odds ratio (OR) with 95% confidence interval (95%CI) and P-value, and those of continuous data are presented as the mean difference (MD) with 95%CI and P-value. In addition, the procedure time of two studies [8, 9], which was previously shown as median and interquartile range (IQR), was estimated and transformed to mean and standard deviation (SD) on the utilization of the conversion method proposed by Shi et al. [10].

This meta-analysis was conducted using two models: the fixed-effect model and the random-effect model. The fixed-effect model was applied to outcomes without significant heterogeneity while the random-effect model was applied to significant heterogeneity. The *I*^*2*^ statistic was utilized to assess heterogeneity among the included studies. *I*^*2*^ values >50% indicate significant heterogeneity. Sensitivity analysis was used for identifying the source of significant heterogeneity. If the heterogeneity decreased significantly when excluding a particular study, that study was consider to be a source of heterogeneity.

This systematic review and meta-analysis followed the Preferred Reporting Items for Systematic Reviews and Meta-Analyses (PRISMA) guidelines [11]. The checklist details are listed in S2 Table. Subgroups analyses were performed in 10–19 mm and ≥20 mm sessile colorectal polyps. All analyses were carried out by Review Manager 5.3 (The Nordic Cochrane centre, The Cochrane Collaboration, Copenhagen, Denmark). *P*<0.05 was regarded as statistically significant.

### Assessment of risk of bias

The quality assessment of RCTs was conducted using Cochrane risk of bias tool, which is an effective tool for detecting bias of RCTs in the areas of randomization, allocation concealment, blinding, incomplete outcomes and selective reporting [12]. The bias of risk of each domain was rated as high, medium or low. The methodological quality assessment of cohort studies in the areas of study group selection, comparability and outcomes was conducted using the Newcastle‒Ottawa Scale (NOS). When the NOS score of a cohort study is greater than 6, it is considered as a good study. Two reviewers performed the assessment independently, and a third reviewer dealt with any disagreement.

### Publication bias

The OR values of en bloc resection rate, R0 resection rate, complete resection rate, adverse events rate and recurrence rate were taken as the horizontal coordinate, and SE[log(OR)] was taken as the longitudinal coordinate to construct the funnel plot. The symmetry of funnel plots was observed to evaluate publication bias.

### Quality assessment

The quality of evidence presented in this review was assessed by Grading of Recommendations Assessment, Development and Evaluation (GRADE) methodology. Two reviewers judged the inconsistency, risk of bias, indirectness, imprecision and publication bias independently and classified the quality as high, moderate, low or very low.

## Results

### Search results and population characteristics

**Fig 1:**
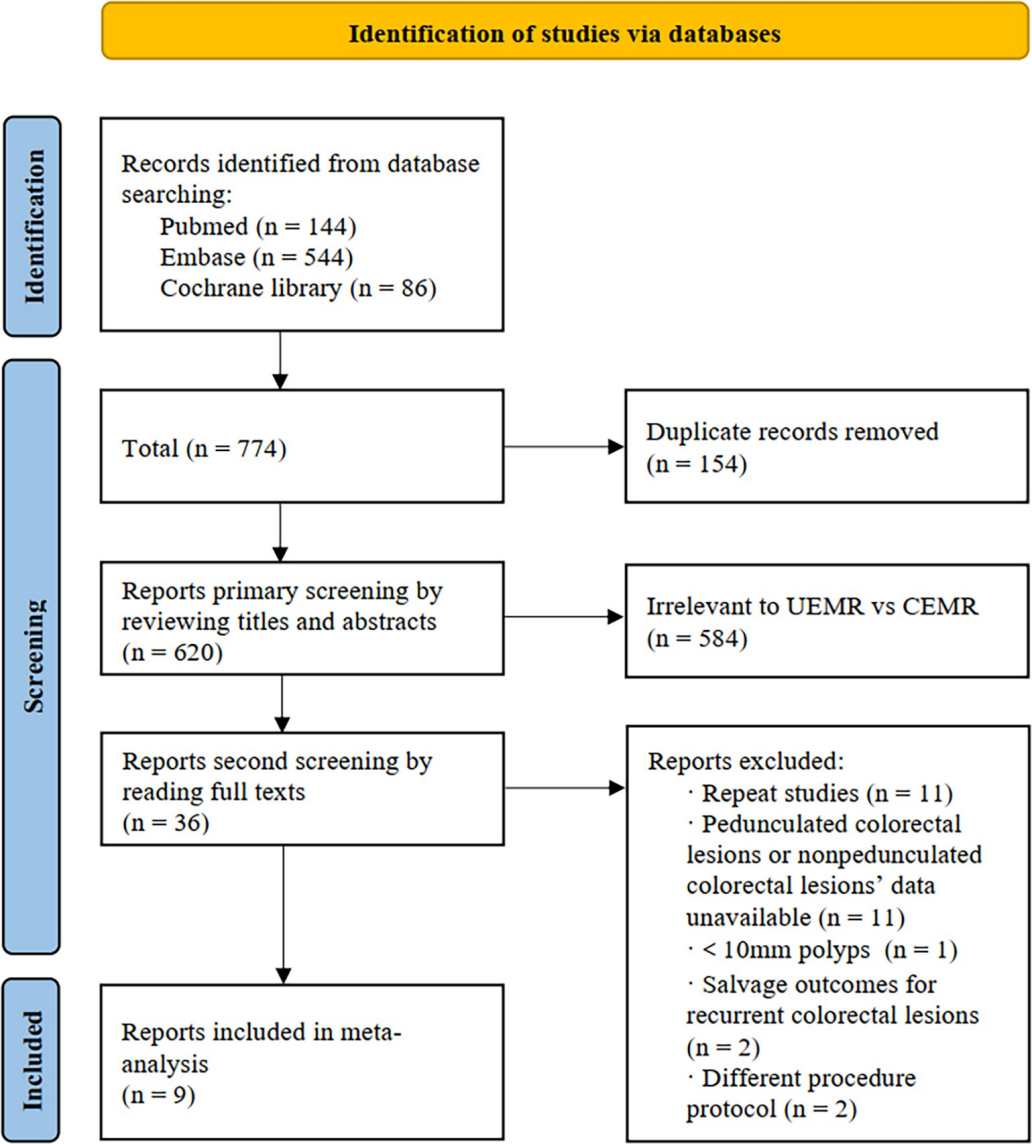
PRISMA flow diagram for screening studies.

There were 774 potentially relevant citations retrieved, of which 620 articles were screened after removing duplicates. By reading the titles and abstracts, 584 articles were identified as irrelevant and removed. Finally, 36 articles were further evaluated by reading through full texts, and 9 articles [5, 6, 8, 9, 13–17] met the inclusion criteria and were included in our analysis, including 6 RCTs [5, 6, 8, 15–17], 2 propensity-score matched cohort studies [13, 14] and 1 retrospective cohort study [9]. Fig 1 shows the study selection process. Among these studies, four were single-centre studies [6, 13–15], and five were multicentre studies [5, 8, 9, 16, 17]. It is worth noting that two studies [9, 15] contained data on 6–9 mm or pedunculated colorectal polyps, but only data on ≥10 mm sessile colorectal polyps were included. Overall, 1,965 patients were involved into this analysis, with 1,285 men and 680 women. The mean age ranged from 64 to 71 years. Among the 1,727 colorectal polyps, 881 polyps were removed by UEMR, and 846 were removed by CEMR. Table 1 shows the study details and population characteristics.

**Table 1:** Study details and characteristics.

Author, year	Study design	No. of patients		Age, years mean (SD)[range]		Sex (Male/Female)		Lesion criteria	No. of lesions		Lesions size Mean (SD) [range]		Lesion location proximal/distal		Primary outcome	Follow-up period
		UEMR	CEMR	UEMR	CEMR	UEMR	CEMR		UEMR	CEMR	UEMR	CEMR	UEMR	CEMR		
Lenz et al, 2022 [16]	RCT, multicentre (2), Brazil, 04/2017-03/2021	53	52	64.4	64.0	25/28	22/30	10–40 mm naïve and nonpedunculated lesions	61	59	median 17.4	median 17.5	41/20	49/10	recurrence rate after 6 months	6 m
Hamerski et al, 2019 (abs) [5]	RCT, multicentre (4), USA 3+Italy 1	158	145	66.5 [39–88]	68.3 [34–90]	77/81	72/73	≥15 mm large colon polyps	158	145	29.5 (10.1)	29.5 (9.1)	–	–	curative resection rate	3–6m
Nagl et al, 2021 [6]	RCT, single-centre, Germany, 08/2017–10/2020	81	76	68.1 (9.6) [33–85]	66.3 (11.9) [38–91]	51/30	52/24	20–40 mm sessile or flat colonic polyps	81	76	27.8 (6.2) [20–40]	28.1 (6.6) [20–40]	70/11	63/13	recurrence rate after 6 months	6 m
Sánchez et al, 2022 [17]	RCT, multicentre (11), Spain, 02/2018-02/2020	145	153	67.5(10.4)	67.2(9.8)	87/58	96/57	>20 mm nonpedunculated polyps	149	162	median 30.0[IQR 25.0–40.0]	median 30.0[IQR 25.0–38.8]	48/101	48/114	recurrence rate during follow-up	6m and 18m
Yen et al, 2020 [15]	RCT, single-centre, USA, 10/2016–09/2018	128	127	64.4 (8.3) [35–81]	64.6 (8.3) [26–78]	123/5	125/2	>5 mm nonpedunculated polyps	68*	50*	9.9 (5.8) [6–40]	9.9 (6.4) [6–45]	202/46	172/42	incomplete resection rate	1d and 30d
Yamashina et al, 2019 [8]	RCT, multicentre (5), Japan, 02/2016–12/2017	108	102	70 [43–86]	68 [42–95]	64/44	75/27	10–20 mm nonpedunculated or sessile polyps	108	102	median 14 [7–25]	median 13.5 [7–25]	66/42	68/34	R0 resection rates	14 d
Nomura et al, 2022 [14]	Retrospective, propensity-score matched study, single-centre, Japan, 01/2018–03/2020	54	54	71 [IQR, 64–75]	67 [IQR, 63–73]	34/20	37/17	10–20 mm nonpedunculated colorectal lesions	54	54	12 [IQR, 10–15]	12 [IQR, 10–15]	37/17	35/19	resection depth	4–7 d
Chien et al, 2019 [13]	Retrospective, propensity-score matched cohort study, single-centre, China, 08/2012–11/2017	121	121	64.1 (12.3)	64.2 (10.0)	79/42	76/45	≥10 mm sessile colon polyps	121	121	17.0 (7.2)	16.6 (6.5)	65/56	70/51	en bloc resection	7–14 d
Cadoni et al, 2018 [9]	Retrospective, multicentre (2), Italy, 01/2015–12/2016	146	141	64.7 (9.0)	65.2 (10.7)	101/45	89/52	≥6 mm sessile, flat and pedunculated polyps	81*	77*	median 10 [IQR, 9.25–15]	median 10 [IQR, 8–15]	–	–	en bloc and R0 resection rates, adverse events	mean 14 m

RCT, Randomized controlled trial; No: number;–, Not reported; UEMR: underwater endoscopic mucosal resection; CEMR: conventional endoscopic mucosal resection; SD: standard deviation.

*: only ≥10 mm and sessile colorectal lesions are included.

### Risk of bias assessment

The Cochrane risk of bias tool and the Newcastle‒Ottawa scale (NOS) were utilized to the quality assessment of RCTs and cohort studies, respectively. All 6 RCTs used block randomization to minimize selection bias [5, 6, 8, 15–17]. The treatment allocation could not be hidden from endoscopists (implementation of UEMR or CEMR), which might increase the risk of performance bias and have an effect on outcome assessment. Although one study was published as a conference abstract, data on outcomes were clearly reported, which diminished the risk of attrition bias [5]. The pre-specified outcomes of each study were reported, which minimizing the reporting bias. S1 Fig illustrates the risk of bias assessment results for enrolled RCTs.

All 3 cohort studies [9, 13, 14] were considered to be of high quality, scoring 7 on the NOS. Although 3 studies were not based on a large population, the cohort size and outcomes of these studies were qualified and clearly reported. Two propensity-score matched studies [13, 14] actively controlled the demographic differences, while another study [9] reported no statistical difference in baseline data between the two groups. Follow-up time was sufficient to assess outcomes of interest, and more than half of the patients participated in it. The quality assessment results for cohort studies are summarized in S3 Table.

### Publication bias

Visual evaluation of the funnel plots was used to identify publication bias. There was no obvious asymmetry in all funnel plots, indicating no publication bias (S2 Fig).

### Grades of evidence levels

Based on the GRADE system, confidence in the estimates was moderate for en bloc resection, R0 resection, complete resection and recurrence rate, low for adverse events (introprocedural bleeding, delayed bleeding, perforation), and very low for procedure time. The elements considered in downgrading the evidence include risk of bias in all results, inconsistency in procedure time and imprecision in adverse events. Details are presented in S4 Table.

### En bloc resection rate

**Fig 2:**
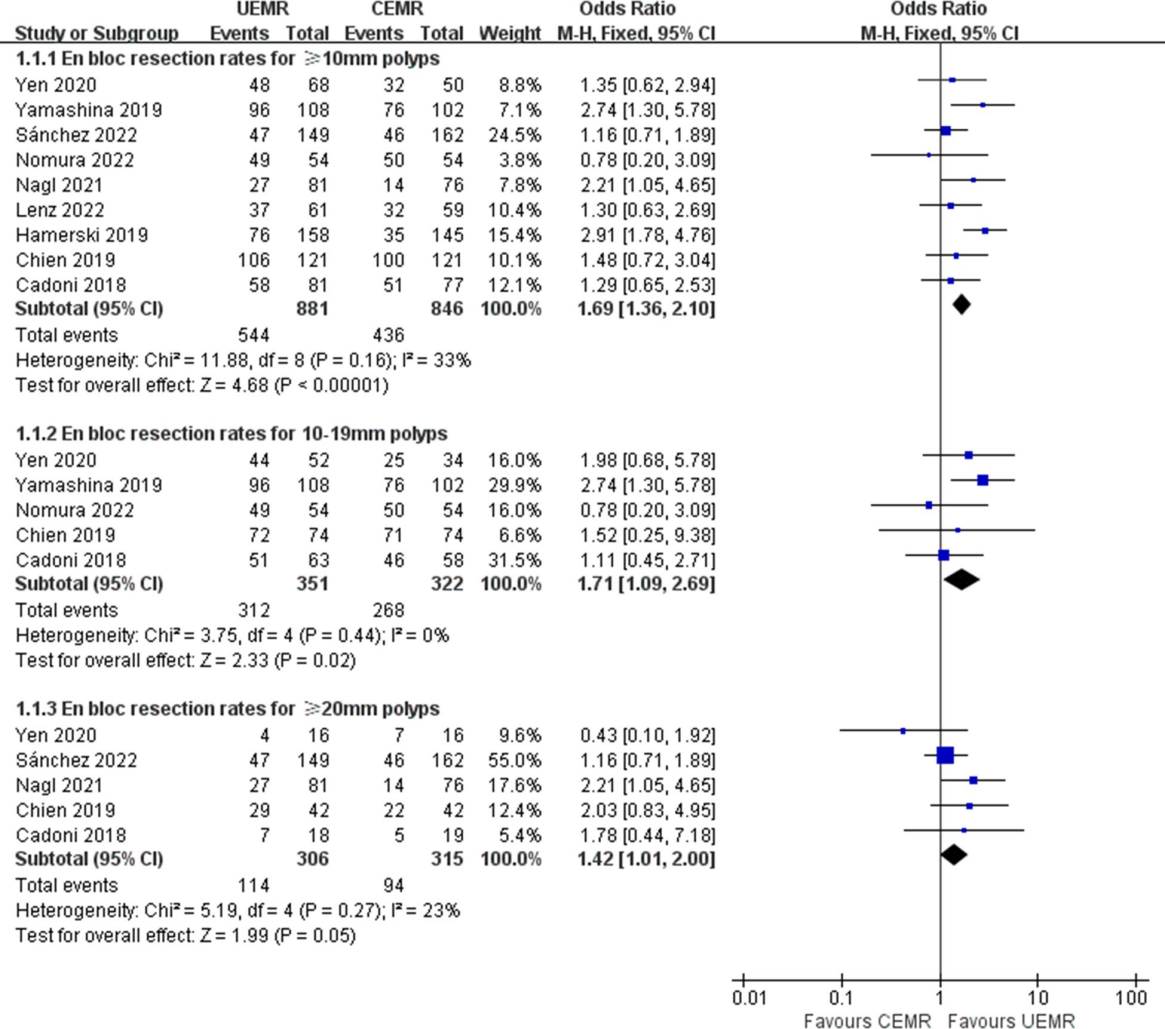
Forest plots of en bloc resection rates in UEMR versus CEMR for sessile colorectal polyps.

All 9 studies reported data on en bloc resection rate [5, 6, 8, 9, 13–17]. In UEMR group, the pooled en bloc resection rate was 61.75%, while it was 51.54% in CEMR group. Compared with CEMR, UEMR was associated with a higher proportion of en bloc resection (OR 1.69, 95%CI 1.36–2.10, *p*<0.00001, *I*^*2*^ = 33%). No significant heterogeneity was found among these studies. Subgroup analysis was conducted based on polyp size. The results showed that for 10–19 mm polyps, the en bloc resection rate of UEMR was greater than that of CEMR (OR 1.71, 95%CI 1.09–2.69, *p* = 0.02, *I*^*2*^ = 0%). However, the en bloc resection rates between the two techniques were comparable for ≥20 mm polyps (OR 1.42, 95%CI 1.01–2.00, *p* = 0.05, *I*^*2*^ = 23%) (Fig 2).

### R0 resection rate

**Fig 3:**
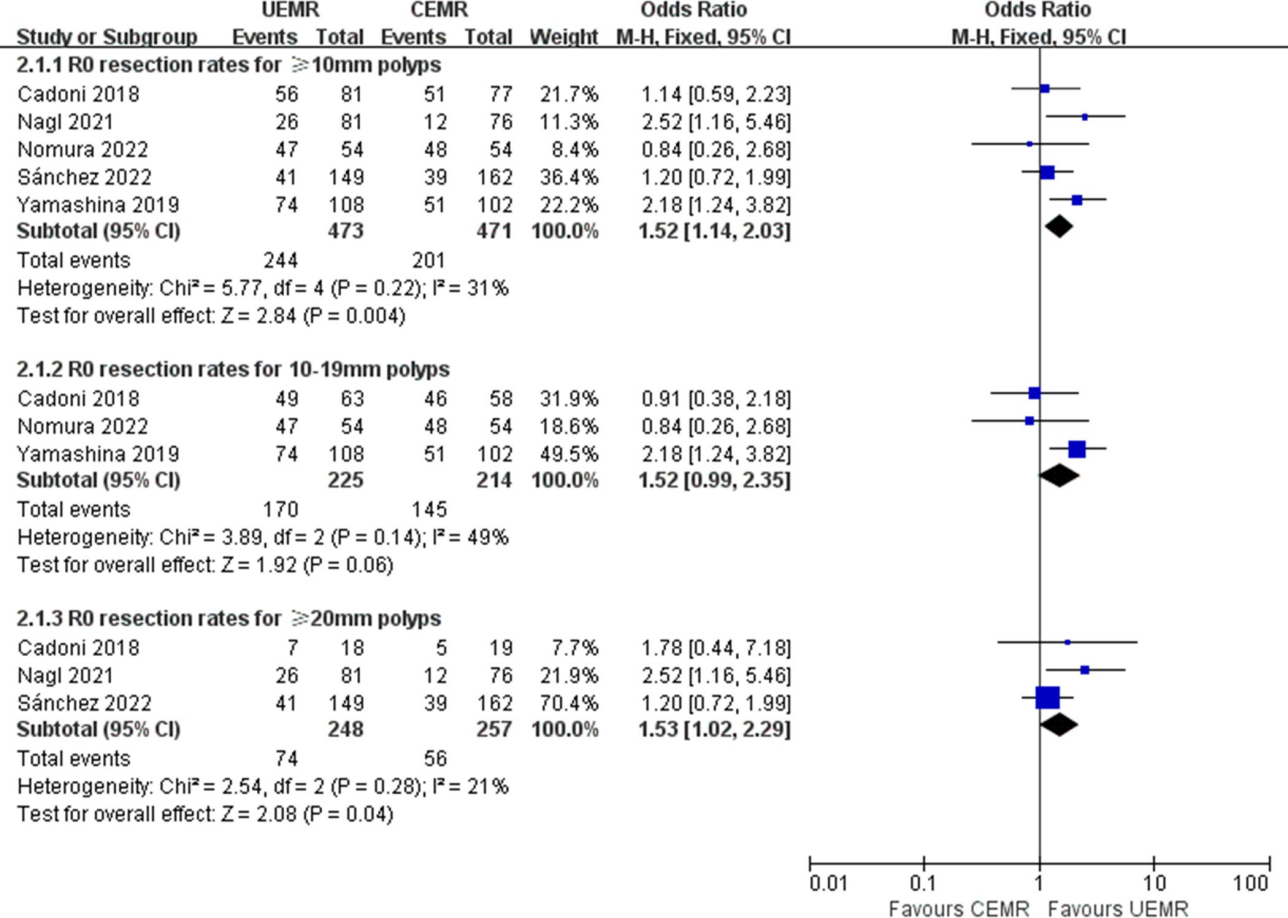
Forest plots of R0 resection rates in UEMR versus CEMR for sessile colorectal polyps.

Five articles reported R0 resection rate [6, 8, 9, 14, 17]. The pooled R0 resection rates were 51.59% with UEMR and 42.68% with CEMR. UEMR was associated with a higher proportion of R0 resection than CEMR (OR 1.52, 95%CI 1.14–2.03, *p* = 0.004, *I*^*2*^ = 31%). Subgroup analysis showed that for polyps with 10–19 mm, UEMR and CEMR had comparable R0 resection rates (OR 1.52, 95%CI 0.99–2.35, *p* = 0.06, *I*^*2*^ = 49%). However, the ≥20 mm polyp subgroup showed a higher R0 resection rate with UEMR (OR 1.53, 95%CI 1.02–2.29, *p* = 0.04, *I*^*2*^ = 21%) (Fig 3).

### Complete resection rate

**Fig 4:**
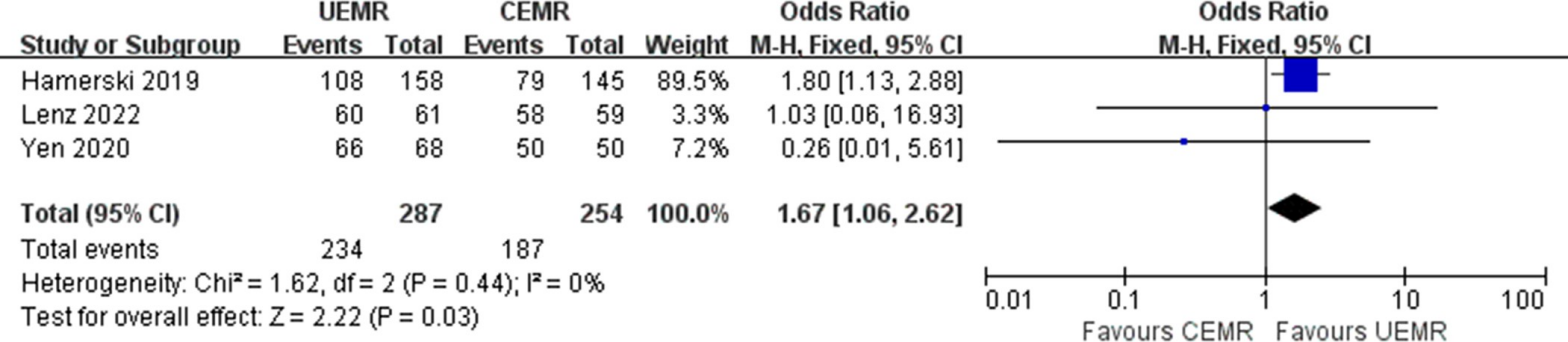
Forest plot of complete resection rates in UEMR versus CEMR for ≥10 mm sessile colorectal polyps.

Three studies reported the results of the complete resection rate [5, 15, 16]. The pooled complete resection rates were 81.53% with UEMR and 73.62% with CEMR. Compared to CEMR, UEMR was associated with higher complete resection rate (OR 1.67, 95%CI 1.06–2.62, *p* = 0.03, *I*^*2*^ = 0%). No significant heterogeneity was found among these studies (Fig 4).

### Procedure time

**Fig 5:**
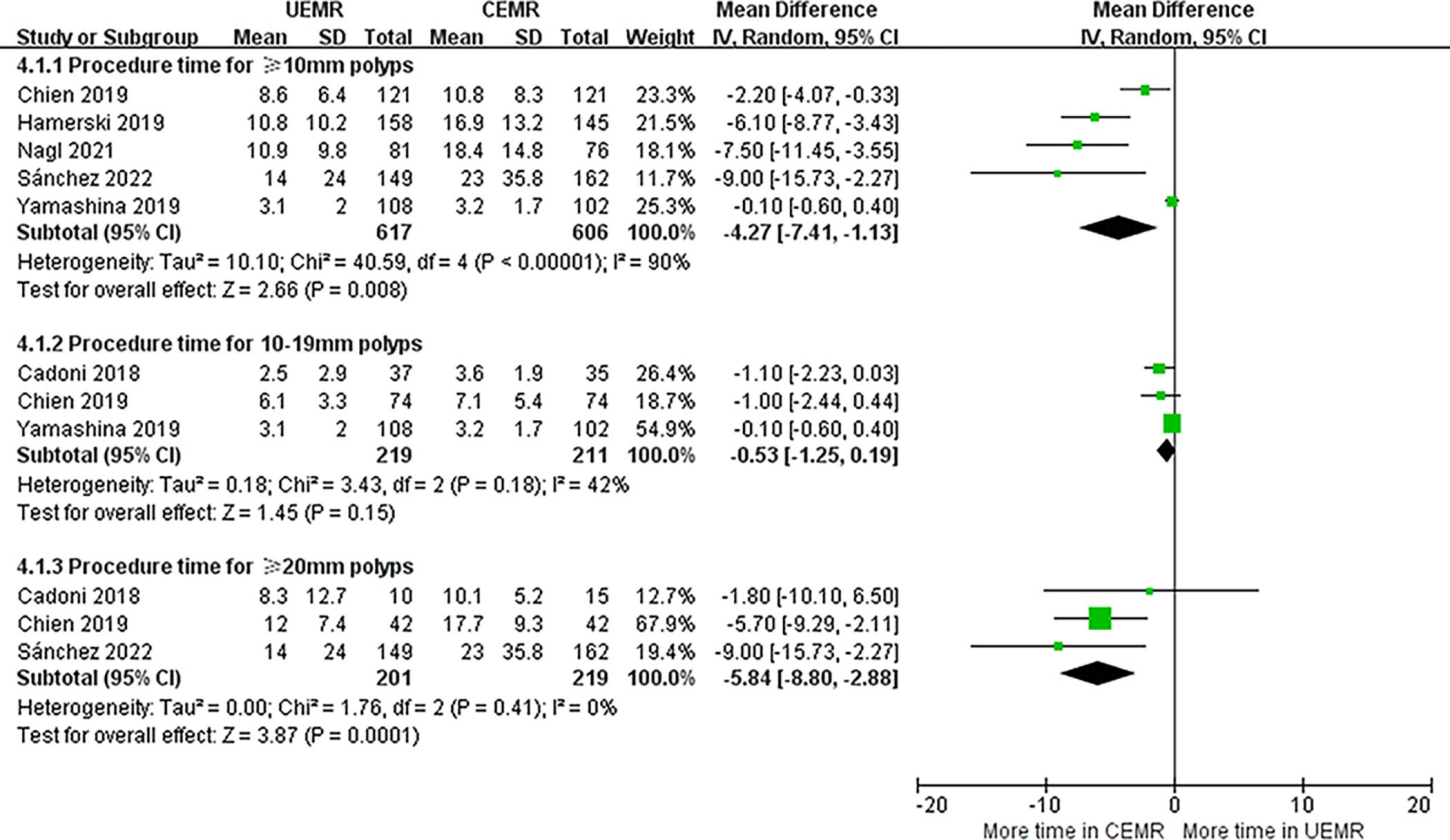
Forest plots of procedure time in UEMR versus CEMR for sessile colorectal polyps.

Five articles provided data on procedure time [5, 6, 8, 13, 17]. There was shorter procedure time in UEMR group than CEMR group (MD ‒4.27, 95%CI ‒7.41 to ‒1.13, *p* = 0.008, *I*^*2*^ = 90%). Significant heterogeneity in procedure time was found between these studies (*I*^*2*^ = 90%). The sensitivity analysis that excluded studies one by one failed to identify a specific article accountable for high heterogeneity. According to subgroup analysis, procedure time spent on UEMR was significantly reduced for ≥20 mm polyps (MD ‒5.84, 95%CI ‒8.80 to ‒2.88, *p* = 0.0001, *I*^*2*^ = 0%) but not for 10–19 mm polyps (MD ‒0.53, 95%CI ‒1.25 to 0.19, *p* = 0.15, *I*^*2*^ = 42%). Furthermore, heterogeneity was reduced in the ≥20 mm polyp subgroup analysis (*I*^*2*^ = 0%), as well as in the 10–19 mm polyp subgroup (*I*^*2*^ = 42%) (Fig 5).

### Adverse events

**Fig 6:**
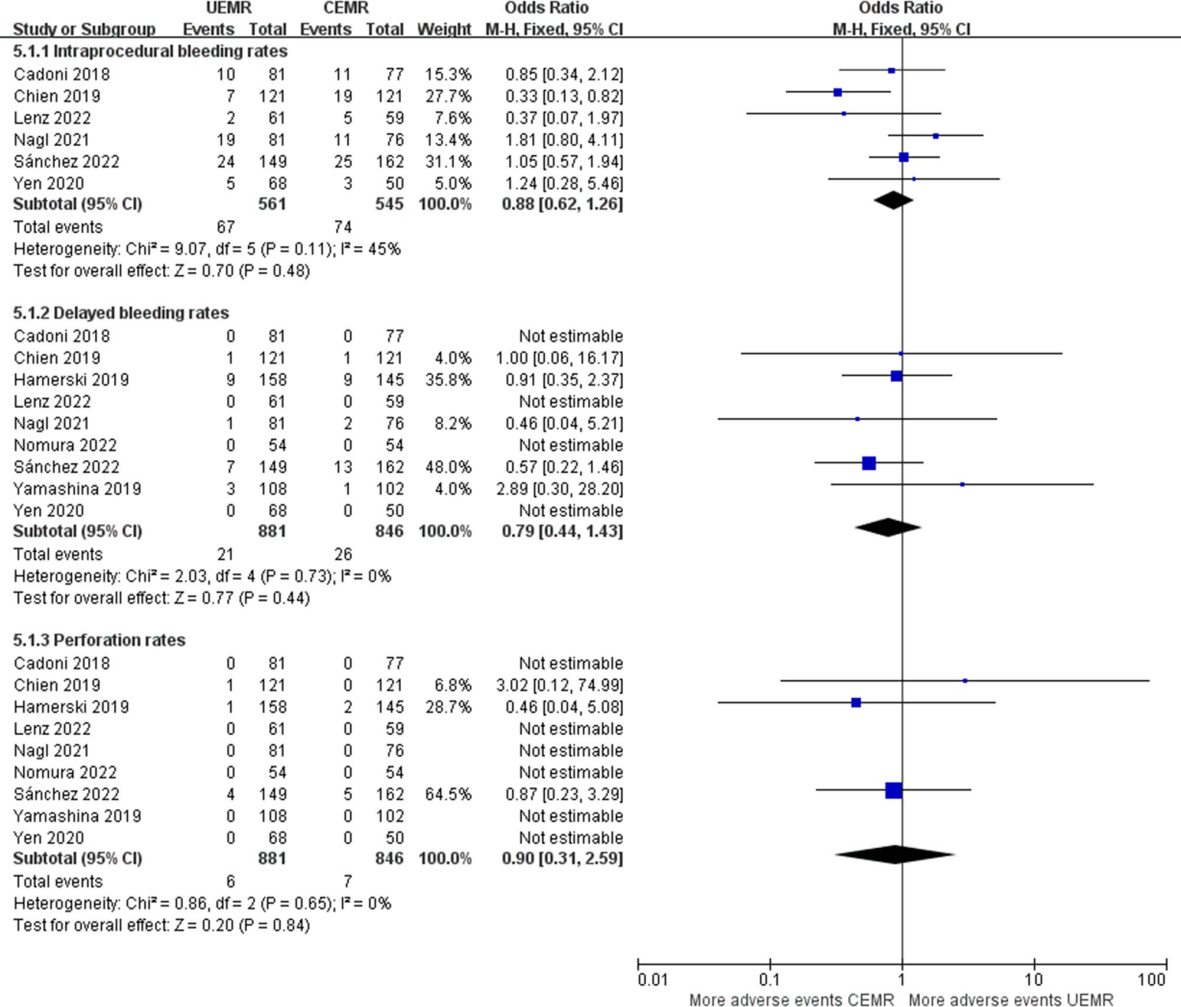
Forest plots of adverse events in UEMR versus CEMR for ≥10 mm sessile colorectal lesions.

Data regarding adverse events were provided by all 9 studies [5, 6, 8, 9, 13–17]. The pooled incidences of intraprocedural bleeding, delayed bleeding and perforation were 11.94%, 2.38% and 0.68% with UEMR and 13.58%, 3.07% and 0.83% with CEMR, respectively. The pooled OR was 0.88 (95%CI 0.62–1.26, *p* = 0.48, *I*^*2*^ = 45%) for intraprocedural bleeding, 0.79 (95%CI 0.44–1.43, *p* = 0.44, *I*^*2*^ = 0%) for delayed bleeding and 0.90 (95%CI 0.31–2.59, *p* = 0.84, *I*^*2*^ = 0%) for perforation (Fig 6). Overall, both techniques had comparable risk of adverse events.

### Recurrence

**Fig 7:**
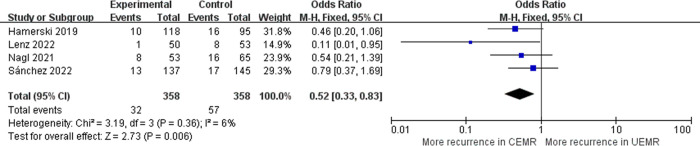
Forest plot of recurrence rates in UEMR versus CEMR for ≥10 mm sessile colorectal lesions.

Four studies examined the recurrence of 716 colorectal polyps [5, 6, 16, 17]. Over at least 3 months of follow-up, UEMR group had a significantly lower recurrence rate than CEMR group (OR 0.52, 95%CI 0.33–0.83, *p* = 0.006, *I*^*2*^ = 6%) (Fig 7). The pooled recurrence rate was 8.94% with UEMR and 15.92% with CEMR. No significant heterogeneity was revealed among these studies.

## Discussion

CEMR is a standard method to remove ≥10 mm sessile colorectal polyps [17]. However, the bowel wall may become thin due to gas insufflation, potentially increasing the risk of transmural burn and perforation [7]. In addition, submucosal injection, an important step to form a cushion and promote safe trapping of lesions, may stretch the originally flat polyps and increase the tissue tension, which would cause the snare to slip off during the closure process and result in piecemeal resection [18]. In 2012, Binmoeller et al. [7] used UEMR instead of CEMR to remove colorectal polyps. As a novel method without submucosal injection, multiple studies were conducted to compare the effectiveness of UEMR versus CEMR. This study is the first systematic review and meta-analysis to investigate the efficiency and safety of UEMR versus CEMR in removing ≥10 mm sessile colorectal polyps. Nine studies were included in our analysis [5, 6, 8, 9, 13–17], with 881 lesions excised by UEMR and 846 by CEMR. As demonstrated by the results, UEMR was superior to CEMR for en bloc resection rate, R0 resection rate, complete resection rate, recurrence rate and procedure time in the treatment of ≥10 mm sessile colorectal polyps, with no significant increase in adverse events.

Failure of en bloc resection means that the lesion needs to be removed in piece, which is related to the high risk of local recurrence [18]. Consistent with the findings of earlier meta-analysis [19], this meta-analysis showed that UEMR was able to achieve higher en bloc resection rate and lower recurrence rate than CEMR for ≥10 mm polyps, without increasing adverse events. In addition, higher R0 resection rate and complete resection rate with UEMR in our results would leave fewer residual lesions, which, together with previous results, was beneficial to patients. Notably, in UEMR group, the improvement of en bloc resection rate was mainly driven by the result of the 10–19 mm polyp subgroup while that of the R0 resection rate was mainly driven by the result of the ≥20 mm polyp subgroup. In fact, the periphery of polyps is enhanced underwater in visualization because of the magnification effect of water, facilitating a more comprehensive assessment of the boundary. The buoyancy effect of water keeps polyps floating in the water and tending to contract, which makes it easier for sessile polyps to be captured and removed with a larger mucosal surface area [7]. Therefore, UEMR has the ability to increase the en bloc resection rate, R0 resection rate, complete resection rate, and thus decrease the recurrence rate.

UEMR could consume shorter procedure time than CEMR, and the improvement was also driven by the subgroup effect of ≥20 mm polyps, suggesting that UEMR may be more efficient for large lesions. The absence of submucosal injection may be one of the explanations. The CEMR procedure is time-consuming. It usually involves determining the appropriate submucosal plane to inject in, injecting in several areas to optimize the position of polyps, and then replacing needles with traps-. On the contrary, the UEMR procedure only requires pumping gas out and filling the lumen with water. Furthermore, water immersion can minimize luminal distension, flexure angulation and loop formation, making the endoscope more manoeuvrable [20]. For all these reasons, UEMR tends to be more time-saving. Substantial heterogeneity was found in the pooled procedure time analysis, which may be due to the different lesion sizes because of less heterogeneity in subgroups based on size.

Despite the absence of submucosal injection, UEMR is as safe as CEMR. Due to the heat sink effect, water could prevent mucosal thermal injury and perforation. Tseng et al. [21] simulated the UEMR procedure with porcine colon and found that the temperature rise on the colonic serosal side of the water group was lower than that of the air group (1.4°C vs. 6.1°C, *p* = 0.004). In addition, endoscopic ultrasound has confirmed that the mucosa and submucosa look like floating in water, while the muscularis propria keeps round and stays away from them, which will minimize the risk of adverse events [7]. In line with the results of previous meta-analyses [19, 22], this study demonstrated that the incidence of adverse events (including intraprocedural bleeding, delayed bleeding and perforation) in the UEMR group was almost on par with that in the control group, strongly supporting the safety of UEMR.

Japan Gastroenterological Endoscopy Society (JGES) recommends endoscopic submucosal dissection (ESD) rather than EMR for removing >20 mm polyps with Paris Ⅱa or Ⅱc morphology or any >30 mm polyps [23]. However, ESD is technically demanding and has a long learning curve [24], while UEMR can easily be learned by endoscopists who are already proficient in CEMR [25]. Although our results showed some advantages with UEMR in resecting ≥20 mm polyps, the difference in effectiveness between UEMR and ESD is unclear. The possible advantages of UEMR in removing large colorectal polyps needs to be further evaluated by more RCTs.

The current study has several advantages. This is the first systematic review and meta-analysis to comprehensively compare the efficiency and safety of UEMR versus CEMR in removing ≥10 mm sessile colorectal polyps. Only RCTs or cohort studies were included to ensure the data quality. According to the Cochrane risk of bias tool and NOS, all enrolled studies were of high quality. In addition, for an accurate representation of EMR in real-world application, we only evaluated polyps ≥10 mm in size. Furthermore, we conducted subgroup analysis based on polyp size to refine the role of UEMR in removing sessile colorectal polyps. Finally, the included studies were conducted in different geographical locations (Japan, Germany, USA, Italy, China, Spain and Brazil), making the results more general applicable.

This study has several limitations. First, the pooled results for procedure time showed considerable heterogeneity, which may be related to various definitions of procedure time besides polyp size. The start points of the UEMR procedure time were water immersion [5, 6, 8] or lesion perimeter marking [9, 17], while the end points of the procedure time were complete removal of polyps [6, 8] or management of immediate complications [9] or closure of mucosal defects with haemoclips [5]. Furthermore, subgroup analyses on the polyp location, morphology and histology between two groups were absent due to the paucity of partial data. Finally, bias associated with performance and outcome detection were present in all included trials because endoscopists could not be ignorant of the treatment method. This is an inherent and inevitable limitation for studies involving novel endoscopic techniques. Measuring results objectively would limit the influence of these biases.

## Conclusion

In conclusion, this systematic review and meta-analysis demonstrates that UEMR is an efficient and safe alternative to CEMR in removing ≥10 mm sessile colorectal polyps. More RCTs are needed to verify the advantages of UEMR over CEMR to promote its application in daily clinical practice.

## Supporting information

S1 Table Search strategy. (PDF)

S2 Table PRISMA 2020 checklist. (PDF)

S3 Table Newcastle-Ottawa Scale. (PDF)

S4 Table Quality of evidence rating by the GRADEpro tool. (PDF)

S1 Fig Cochrane risk of bias assessment in randomized controlled trials. (TIF)

S2 Fig Funnel plots of main results. En bloc resection rate (A), R0 resection rate (B), complete resection rate (C), procedure time (D), intraprocedural bleeding rate (E), delayed bleeding rate (F), perforation rate (G) and recurrence rate (H) in UEMR versus CEMR for ≥10 mm sessile colorectal polyps. *SE*, standard error; *OR*, odds ratio; *MD*, mean difference.
(TIF)
