# Supplementary material for: Underwater versus conventional endoscopic mucosal resection for ≥10 mm sessile or flat colorectal polyps: A systematic review and meta-analysis
Source: PLoS One. 2024 Mar 7;19(3):e0299931. doi: 10.1371/journal.pone.0299931 (PMC10919657; doi:10.1371/journal.pone.0299931)
Supplement: S1 Table — (PDF) [file pone.0299931.s001.pdf]

S1 Table. Search strategy

Search strategy in Pubmed (Data run: 11/2/2023)		
**Number**	**Query**	**Results**
**#1**	(("resection"[Title/Abstract] OR "mucosectomy"[Title/Abstract]) AND "endoscop*"[Title/Abstract]) OR "endoscopic mucosal resection"[MeSH Terms] OR "EMR"[Title/Abstract]	39,163
**#2**	"underwater"[Title/Abstract] OR "water"[Title/Abstract]	945,545
**#3**	"colon*"[Title/Abstract] OR "colorectal"[Title/Abstract] OR "rectum"[Title/Abstract] OR "rectal"[Title/Abstract] OR "intestin*"[Title/Abstract]	1,114,320
**#4**	"polyp*"[Title/Abstract] OR "lesion*"[Title/Abstract] OR "adenoma*"[Title/Abstract] OR "neoplasm*"[Title/Abstract] OR "tumor*"[Title/Abstract] OR "tumour*"[Title/Abstract] OR "carcinom*"[Title/Abstract] OR "cancer*"[Title/Abstract]	4,638,368
**#5**	#1 AND #2 AND #3 AND #4	144

Search strategy in Embase (Data run: 11/2/2023)		
**Number**	**Query**	**Results**
**#1**	'endoscopic mucosal resection'/exp	8,503
**#2**	resection	543,151
**#3**	mucosectomy	1,516
**#4**	endoscop*	577,637
**#5**	#2 OR #3	543,932
**#6**	#4 AND #5	77,152
**#7**	emr	24,151
**#8**	#1 OR #6 OR #7	95,929
**#9**	underwater	12,308
**#10**	water	1,422,482
**#11**	#9 OR #10	1,430,360
**#12**	colon* OR colorectal OR rectum OR rectal OR intestin*	2,113,949
**#13**	polyp* OR lesion* OR adenoma* OR neoplasm* OR tumor* OR tumour* OR carcinom* OR cancer*	8,223,650
**#14**	#8 AND #11 AND #12 AND #13	544

Search strategy in Cochrane Library(Data run: 11/2/2023)		
**Number**	**Query**	**Results**
**#1**	MeSH descriptor: [Endoscopic Mucosal Resection] explode all trees	157
**#2**	(resection or mucosectomy):ti,ab,kw	32,407
**#3**	(endoscop*):ti,ab,kw	31,649
**#4**	#2 and #3	3,236
**#5**	('endoscopic mucosal resection' or EMR) :ti,ab,kw	1,744
**#6**	#1 or #4 or #5	4,172
**#7**	(water or underwater):ti,ab,kw	36,067
**#8**	(colon* or colorectal or rectum or rectal or intestin*):ti,ab,kw	84,943
**#9**	(polyp* or lesion* or adenoma* or neoplasm* or tumor* or tumour*	295,020

	or carcinom* or cancer*):ti,ab,kw	
**#10**	#6 and #7 and #8 and #9	86
