# Supplementary material for: Underwater versus conventional endoscopic mucosal resection for ≥10 mm sessile or flat colorectal polyps: A systematic review and meta-analysis
Source: PLoS One. 2024 Mar 7;19(3):e0299931. doi: 10.1371/journal.pone.0299931 (PMC10919657; doi:10.1371/journal.pone.0299931)
Supplement: S3 Table — (PDF) [file pone.0299931.s003.pdf]

S3 Table. Newcastle-Ottawa Scale: cohort study quality assessment

Study	Selection				Comparability	Outcome			Score	quality
	Representativeness of the average adult in community	Cohort size	Information on clinical outcomes	Outcome not present at start	Factors comparable between the groups	Adequate clinical assessment	Follow up time	Adequacy of follow-up	Max=8	>6: High; 4–6: medium; ≤3: low
	1: population based; 0.5: multicenter; 0: single center	1: >40 patients; 0.5: 20–39; 0: <20	1: information with clarity; 0.5: information derived from percentage value; 0: unclear	1: not present; 0: present	1: yes; 0: no	1: yes; 0: no	1: yes; 0: not mentioned	1: all patients followed up; 0.5: >50% followed up; 0: <50% followed up or not mentioned		
Nomura 2022	0	1	1	1	1	1	1	1	7	high
Chien 2019	0	1	1	1	1	1	1	1	7	high
Cadoni 2018	0.5	1	1	1	1	1	1	0.5	7	high
