# Supplementary material for: Underwater versus conventional endoscopic mucosal resection for ≥10 mm sessile or flat colorectal polyps: A systematic review and meta-analysis
Source: PLoS One. 2024 Mar 7;19(3):e0299931. doi: 10.1371/journal.pone.0299931 (PMC10919657; doi:10.1371/journal.pone.0299931)
Supplement: S4 Table — (PDF) [file pone.0299931.s004.pdf]

S5 Table. Quality of evidence rating by the GRADEpro tool

Underwater versus conventional endoscopic mucosal resection for ≥10mm sessile colorectal polyps										
**Outcome**	**Quality assessment**					**No of lesions in UEMR**	**No of lesions in CEMR**	**Effect**		**Quality**
	**Risk of bias**	**Inconsistency**	**Indirectness**	**Imprecision**	**Other considerations**			**Relative (95% CI)**	**Absolute**	
**En bloc resection rates**	serious[1]	no serious	no serious	no serious	none	544/881 (61.7%)	436/846 (51.5%)	OR 1.69 (1.36 to 2.1)	127 more per 1000 (from 76 more to 175 more)	⊕⊕⊕O MODERATE
**R0 resection rates**	serious[1]	no serious	no serious	no serious	none	244/473 (51.6%)	201/471 (42.7%)	OR 1.52 (1.14 to 2.03)	104 more per 1000 (from 32 more to 175 more)	⊕⊕⊕O MODERATE
**Complete resection rates**	serious[1]	no serious	no serious	no serious	none	234/287 (81.5%)	187/254 (73.6%)	OR 1.67 (1.06 to 2.62)	87 more per 1000 (from 11 more to 143 more)	⊕⊕⊕O MODERATE
**Procedure time**	serious[1]	very serious[2]	no serious	no serious	none	617	606	–	MD 4.27 lower (7.41 to 1.13 lower)	⊕OOO VERY LOW
**Intraprocedural bleeding rates**	serious[1]	no serious	no serious	serious[3]	none	67/561 (11.9%)	74/545 (13.6%)	OR 0.88 (0.62 to 1.26)	14 fewer per 1000 (from 47 fewer to 29 more)	⊕⊕OO LOW
**Delayed bleeding rates**	serious[1]	no serious	no serious	serious[3]	none	21/881 (2.4%)	26/846 (3.1%)	OR 0.79 (0.44 to 1.43)	6 fewer per 1000 (from 17 fewer to 13 more)	⊕⊕OO LOW
**Perforation rates**	serious[1]	no serious	no serious	serious[3]	none	6/881 (0.7%)	7/846 (0.8%)	OR 0.9 (0.31 to 2.59)	1 fewer per 1000 (from 6 fewer to 13 more)	⊕⊕OO LOW
**Recurrence rates**	serious[1]	no serious	no serious	no serious	none	32/358 (8.9%)	57/358 (15.9%)	OR 0.52 (0.33 to 0.83)	70 fewer per 1000 (from 23 fewer to 101 fewer)	⊕⊕⊕O MODERATE

1 This domain was downgraded by 1 point because there was evidence of performance and detection bias in all studies included in this review.

2 This domain was downgraded by 2 points because there was evidence of heterogeneity (I^2 > 75%) in this outcome.

3 This domain was downgraded by 2 points because there was no statistically significant difference in this outcome.
