# Supplementary figures and images for: Underwater versus conventional endoscopic mucosal resection for ≥10 mm sessile or flat colorectal polyps: A systematic review and meta-analysis
Source: PLoS One. 2024 Mar 7;19(3):e0299931. doi: 10.1371/journal.pone.0299931 (PMC10919657; doi:10.1371/journal.pone.0299931)

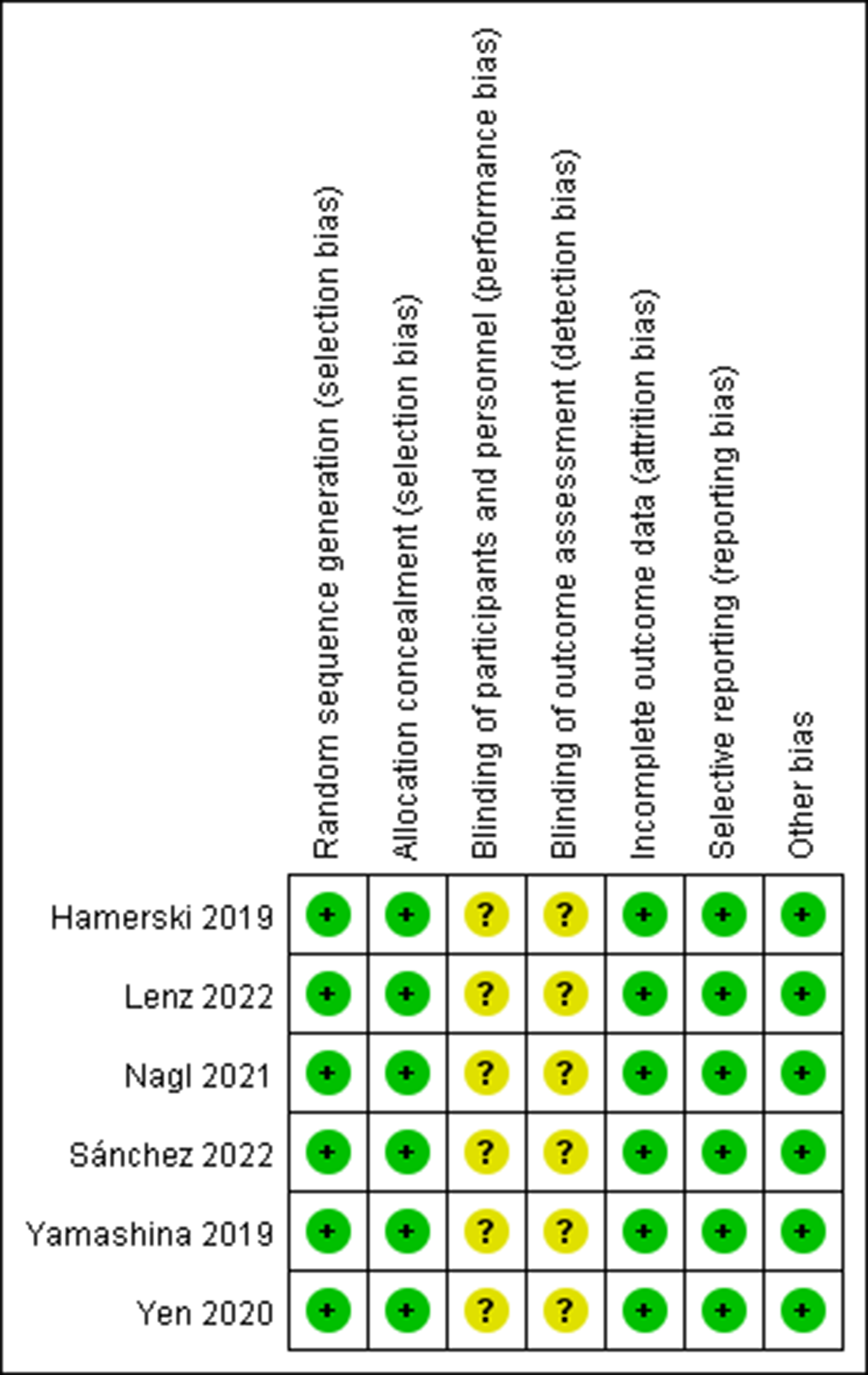

Supplement: S1 Fig — (TIF) [file pone.0299931.s005.tif]
